# Misuse of Iron Preparations

**Published:** 1880-11

**Authors:** Alf. W. Perry


### MISUSE OF IRON PREPARATIONS.

In cases of debility, prostration, or loss of appetite, preparations of iron, alone or variously combined with bitter tonics, are seemingly indicated clearly, and are very generally used. But in many cases they do harm, either from their being administered at a wrong time or because they are not tolerated under any form or circumstance. The greatest abuse of iron is where it is given for loss of appetite or difficult digestion, and when it is given within half an hour before eating or within three hours after. We have found entirely to our own satisfaction, both by clinical observation and by experiment, that iron preparations introduced into the stomach while digestion is going on either hinder or arrest the process. Our attention was first called to this by clinical observation. We treated a patient some time since, who had been treated by another physician for slight cystitis with considerable depression of spirits and loss of appetite. The man was, however, quite strong, and not emaciated. He also had a slight feeling of weight after eating. For this he was given 6 gr. doses of cit. ferri. et quiniæ a half hour before eating. This made the feeling of weight worse. The physician increased the dose to 10 grs., and it was still worse, and the appetite also remained very small, with considerable depression of spirits. Upon taking charge of the case and finding out what he had been taking, I stopped all tonics and used only slightly anodyne suppositories for the irritable bladder. His appetite immediately improved, and the weight at the epigastrium disappeared. This is only one of many cases where we have found the use of iron preparations to produce indigestion, feeling of fullness at the epigastrium, and even vomiting when given at the wrong time. To find reason for this we tried the effects of iron preparations upon artificial digestion of animal substances.

We took two test tubes, and put in each the same quantity of artificial gastric juice, i. e., a two per cent. mixture of hydrochloric acid with pepsin, and then placed in each a few pieces of coagulated albumen. Into one was also placed ʒ i of elixir of iron and quinia. Both test tubes were kept at blood-heat for ten hours. In four hours the albumen was rapidly disintegrating in the gastric juice alone. At the same time the albumen in the tube containing gastric juice and iron was apparently intact.

In ten hours the albumen in the gastric juice alone was entirely dissolved, while in the other tube it was still intact.

In a number of experiments the same results were arrived at. We may then consider it established that some iron preparations, if not all, when taken into the stomach during digestion, hinder that act, and therefore they should not be given so that they will then be present in the stomach. Whether the secretion of gastric juice is affected by iron preparations or other tonics it will remain for further observation to determine, and thus contribute valuable addition to therapeutics. There are many medicines the effects of which, whether good, bad or nil, depend entirely on the time of administration.

The tendency both on the part of prescribers and the large drug manufacturers is to combine iron with other tonics, so that the markets are flooded with elixirs, syrups, and wines of iron and quinine, iron and strychnia, iron, strychnia and pepsin, and so on *ad infinitum.* The combinations with pepsin are a shameful waste of this valuable medicine, and well calculated to bring it into disrepute. None of the others above mentioned should be used for or in any gastric derangement, except with due regard to time of administration. The most suitable time to give iron is one hour before meals or four hours afterward.
—*Alf. W. Perry, M. D., in Western Lancet.*

